# Correlation of Superlattice Cross-Plane Thermal Conductivity with Emission Wavelength in InAlAs/InGaAs Quantum Cascade Lasers

**DOI:** 10.3390/mi13111934

**Published:** 2022-11-09

**Authors:** Alejandro M. Villalobos Meza, Monas Shahzad, Dagan Hathaway, Hong Shu, Arkadiy Lyakh

**Affiliations:** 1 NanoScience Technology Center, University of Central Florida, 12424 Research Pkwy, Orlando, FL 32816, USA; 2 College of Optics and Photonics, University of Central Florida, 4304 Scorpius St., Orlando, FL 32816, USA; 3 IRGLARE, LLC, 3259 Progress Drive, Orlando, FL 32826, USA; 4 Department of Physics, University of Central Florida, 4111 Libra Dr., Orlando, FL 32816, USA

**Keywords:** quantum cascade lasers, thermal conductivity, infrared

## Abstract

The low cross-plane thermal conductivity of quantum cascade lasers (QCLs) is a significant limitation in their Continuous-Wave (CW) performance. Structural parameters such as individual layer thicknesses and interface density vary for QCLs with different target emission wavelengths, and these design parameters are expected to influence the cross-plane thermal conductivity. Though previous works have used theoretical models and experimental data to quantify thermal conductivity, the correlation between target wavelength and thermal conductivity has yet to be reported for QCLs. In this work, we observe a general trend across a group of QCLs emitting from 3.7 to 8.7 µm: as the QCL design changes to reduce wavelength, the thermal conductivity decreases as well. Numerically, we measured an approximate 70% reduction in thermal conductivity, from 1.5 W/(m·K) for the 8.7 µm device, to 0.9 W/(m·K) for the 3.7 µm device. Analysis of these structures with the Diffuse Mismatch Model (DMM) for thermal boundary resistance (TBR) shows that the largest contribution of this effect is the impact of superlattice interface density on the thermal conductivity. The observed changes in conductivity result in significant changes in projected CW optical power and should be considered in laser design.

## 1. Introduction

Quantum Cascade Lasers (QCLs) are infrared semiconductor laser devices that operate through electronic inter-sub-band transitions in a superlattice core, which boast numerous commercial and defense applications. A well-reported limitation of the room-temperature operation of QCLs is poor heat dissipation in the laser core, which hinders Continuous-Wave (CW) performance [1,2,3,4]. This limitation is partially caused by the anisotropic thermal conductivity of the laser core, which has been reported to be significantly lower in the cross-plane direction due to the laser core being composed of up to one thousand individual ternary semiconductor layers [1,2,3,4,5]. This is exacerbated by the high input power density typical of QCLs [1,2,3,4,5,6]. QCLs operate in a wide range of wavelengths, from mid-infrared (MIR) to Terahertz (THz) wavelength regions [7,8,9], and a relevant aspect of QCL design primarily considers the modification of structural parameters to achieve the desired emission wavelength. It is important to consider how these changes in QCL structure affect the thermal conductivity [5] when optimizing QCLs for CW performance.

The superlattice for state-of-the-art QCLs is typically composed of InAlAs barriers and InGaAs quantum wells [4,10]. The reduction in emission wavelength requires employment of narrower quantum wells to increase laser transition energy, and the InGaAs barrier is known to have a higher bulk conductivity (5.0 W/(m·K)) than that of the InAlAs wells (2.5 W/(m·K)) [5,11]. Therefore, the weighted average thermal conductivity is generally lower for shorter wavelength devices. The reduction in quantum well thickness also increases the superlattice interface density, hindering phonon transport across the superlattice and further reducing cross-plane thermal conductivity [5]. Consequently, it is expected that, as the target emission wavelength decreases, the cross-plane thermal conductivity will decrease in turn due to changes in laser design. The goal of this work is to quantify this effect.

Thermal characteristics of QCL structures have been extensively studied [1,2,4,5,6,7,8,9,12] through a variety of experimental and theoretical methods, lending to the significance of the thermal transport problem. Additionally, recent studies have demonstrated a variety of approaches for determining the temperature profile of quantum cascade lasers [13,14,15,16]. Through similar methods, the relationship between thermal conductivity and wavelength, which to our knowledge has yet to be probed for mid-infrared (MIR) and long-wave infrared (LWIR) QCLs, can be quantified. This is done namely with an experimental approach as in [15,16], and through a theoretical model as in [3,13]. We can confirm the trend in both cases and are able to present the significance of the interface density in the reduction of thermal conductivity, as well as demonstrate how this reduced conductivity affects CW performance.

## 2. Materials and Methods

The devices considered were all buried-heterostructure InP-based quantum cascade lasers whose core was composed of an InGaAs/InAlAs superlattice. Four different laser core designs were considered for this analysis: two emitting at 3.7 µm (S25 and D41), another emitting at 5.7 µm (S1), and a fourth long-wave device emitting at 8.7 µm (S12). For each structure, laser chips were mounted epi-side down onto ceramic AlN sub-mounts and coated with a highly reflective coating. The lasers were operated in CW at 15 °C using a chiller to maintain heat sink temperature. A twofold analysis was conducted to determine the cross-plane thermal conductivity theoretically and experimentally.

### 2.1. Theoretical Approach

A theoretical model based on phonon transport in semiconductor superlattices, which was previously used for Terahertz QCLs [2], was used to project changes in cross-plane thermal conductivity. This model considers the impact of the thermal resistance at the interfaces of the superlattice. This consideration is necessary to calculate the thermal conductivity in the cross-plane direction, and comes in the form of an additional term called the thermal boundary resistance (TBR). TBR is a measure of the heat flow between two solid interfaces of different material properties [17,18,19]. The effective thermal conductivity is calculated as a weighted sum of the thermal conductivities of the bulk constituents of the superlattice [3,13,19]. This is a series summation in the in-plane direction, but when modeling thermal conductivity in the cross-plane direction, the summation must be done in parallel, and the TBR term must be considered:

(1) $$k_{∥}=\frac{L_{1}}{L}k_{1}+\frac{L_{2}}{L}k_{2}$$

(2) $$\kappa_{⊥}^{-1}=\frac{L_{1}}{L}k_{1}^{-1}+\frac{L_{2}}{L}\kappa_{2}^{-1}+\frac{N}{L}*R^{\left(ave\right)}$$

where $L_{i}$ is the total thickness of the *i_th_* material in a single period or “stage” of the superlattice, $k_{i}$ is the thermal conductivity of the *i_th_* material in the superlattice, *L* is the total thickness of a single stage, *N* is the number of interfaces in said stage, and *R^(ave^*^)^ is the average of the thermal boundary resistance of heat flow in both directions of the solid–solid interface [2]. TBR is a material parameter dependent on the mechanical properties of the bulk constituent materials, namely the density and acoustic wave speeds, which themselves depend on the composition of the ternary semiconductor materials [6,17,18,19]. The contribution to the conductivity is itself weighted based on the density of interfaces in a single stage. Table 1 outlines the relevant parameters for the four devices.

The above parameters are not only necessary to calculate the TBR and the thermal conductivity, but are also used to model the geometry of the structure for later 3D thermal simulations. The TBR is taken to be the average of the TBRs for both directions of heat flow.

(3) $$R^{\left(ave\right)}=\frac{R_{1\to 2}+R_{2\to 1}}{2}\;$$

Here *R_i→j_* is the TBR for heat flow from material *i* to material *j* in units of m^2^K/W

(4) $$R_{i\to j}={\left[\frac{1}{2}*\underset{j}{\sum }\nu_{1,j}\Gamma_{1,j}\int_{0}^{\omega_{D}}\hbar \omega \frac{dN_{1,j}\left(\omega ,T\right)}{dT}d\omega \right]}^{-1}\;$$

where $\nu_{n,j}$ denotes the acoustic phonon velocity, subscripts *n* and *j* denote material number and polarization directions (one longitudinal and two transverse), respectively, and $\omega_{D}$ is the Debye frequency. $N_{n,j}\;$is the density of phonon states and $\Gamma_{n,j}$ is the averaged transmission coefficient. Both are defined below as:

(5) $$N_{1,j}\left(\omega ,T\right)=\frac{\omega^{2}}{2\pi^{2}\nu_{1,j}^{3}\left[\exp\left(\frac{\hbar \omega }{k_{B}T}\right)-1\right]}$$

(6) $$\Gamma_{1,j}=\int_{0}^{\frac{\pi }{2}}\alpha_{1\to 2}\left(\theta ,j\right)\cos\theta \;\sin\theta \;d\theta$$

There are two models for calculating the thermal boundary resistance. These are the acoustic mismatch model (AMM) and diffuse mismatch model (DMM). The DMM only considers the probability that phonons crossing the interface will lose all coherence and retain no information on their previous state. In contrast, the AMM considers the polarization of phonons, and considers them as plane waves propagating through a continuum medium. This distinction can be seen in the calculation of the phonon transmission probability term $\alpha_{1\to 2}$ [2,18,19].

(7) $$\alpha_{1\to 2_{DMM}}=\frac{\sum_{j}\nu_{2,j}^{-2}}{\sum_{n,j}\nu_{n,j}^{-2}},\;\;\;\alpha_{1\to 2_{AMM}}=\frac{4\rho_{1}\nu_{1}\rho_{2}\nu_{2}\cos\theta_{1}\cos\theta_{2}}{{\left(\rho_{1}\nu_{1}\cos\theta_{1}+\rho_{2}\nu_{2}\cos\theta_{2}\right)}^{2}}$$

Here $\rho_{n}$ is the density of the *n_th_* material and $\theta_{n}$ is based on Snell’s Law at the interface. As in [2], it is sufficient to consider the phonon interactions as purely adhering to one of the two models for the purposes of estimating TBR and conductivity. Table 2 illustrates the relevant parameters for calculating TBR as they depend on the Ga/Al distribution of the two superlattice constituents.

### 2.2. Experimental Approach

The anticipated effect on thermal conductivity was experimentally studied here across several buried heterostructure devices. Raman thermometry [15,16] was used to measure active region heating as a function of CW electrical power injected into the laser. As in the experimental setup, discussed in [15], 532 nm laser light was focused with a 50× objective lens into a spot size of ~1.3 μm and power 0.6 W onto the center of the laser core at the front facet. A thermal calibration was conducted to record the linear dependence of the Stokes peak position with temperature, which then allowed us to extract the temperature-current curves from the change in the Stokes peak position with input current. Additionally, the conductivity of the S25 structure was estimated by temperature-dependent CW power projection as in [21].

#### COMSOL Thermal Simulation

The aforementioned experimental procedure allowed us to determine how the temperature in the core changed with input current. To extract the thermal conductivity from these data, a 3D COMSOL study that simulated the heating within the active region resulting from the input electrical power was used to measure the maximum facet temperature as a function of input current. The cross-plane thermal conductivity was treated as the only fitting parameter, allowing us to determine the thermal conductivity from the best fit of the temperature curves generated from COMSOL with the experimental data, as in [15]. Using the conductivity as the independent variable in 2D thermal simulations, the CW optical power was projected and compared to experimental CW Light–Current–Voltage (LIV) curves.

## 3. Results

### 3.1. Theoretical Results

Using the previously mentioned parameters, the TBR and conductivity were calculated for each device using both the DMM and AMM. Additionally, the interface density, which is a critical factor in the cross-plane conductivity, was measured to observe its impact. Interface density was taken to be the number of interfaces in a stage divided by the total stage thickness. Table 3 outlines the results of the calculations.

For the purposes of continued analysis, the DMM was used to estimate the thermal conductivity as it had the most agreement with the measured data, falling within 10–15% of the experimental conductivity values (see below). The DMM was more applicable in the case where interface roughness caused scattering to be primarily diffuse [2,20]. The model showed that, although the number of interfaces and the thickness of the individual layers did generally influence the thermal conductivity in a manner that was expected, interface density proved the most consistent structure parameter that agreed with the trend across the four devices tested.

**Figure 1:**
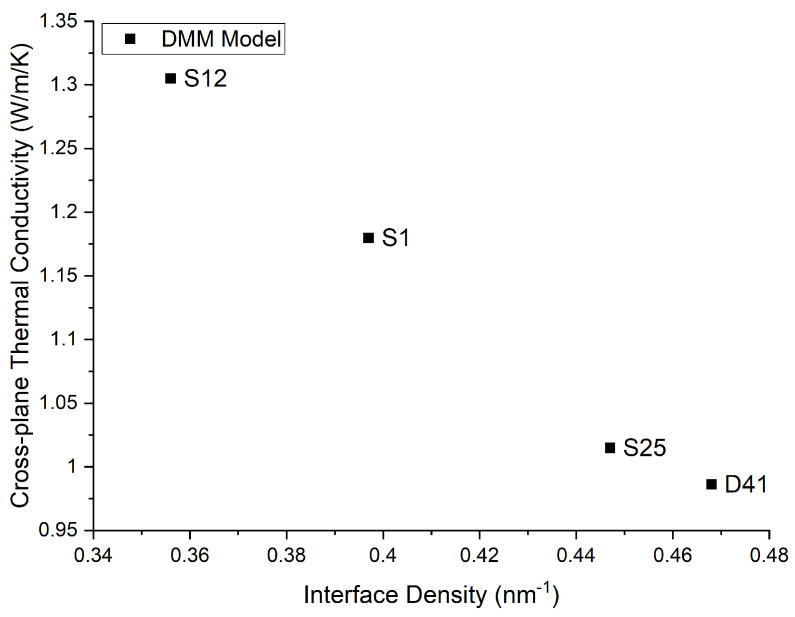
Theoretical relationship between interface density and thermal conductivity as per the DMM.

The relative importance of TBR can be understood by analyzing the value of each term on the right-hand side of Equation (2), summarized in Table 4. We can see that the dominant term in this calculation was the third term, which weighed the TBR based on the interface density. Therefore, interface density was largely responsible for the reduction in conductivity. Figure 1 demonstrates this relationship.

The cross-plane thermal conductivity’s relationship with the interface density was consistent, and as Table 4 shows, this interface density was a direct consequence of the wavelength selection in QCL design. With the theoretical model for conductivity confirming the anticipated relationship between wavelength and conductivity, we could now demonstrate this trend using the experimentally determined conductivities.

### 3.2. Experimental Results

**Figure 2:**
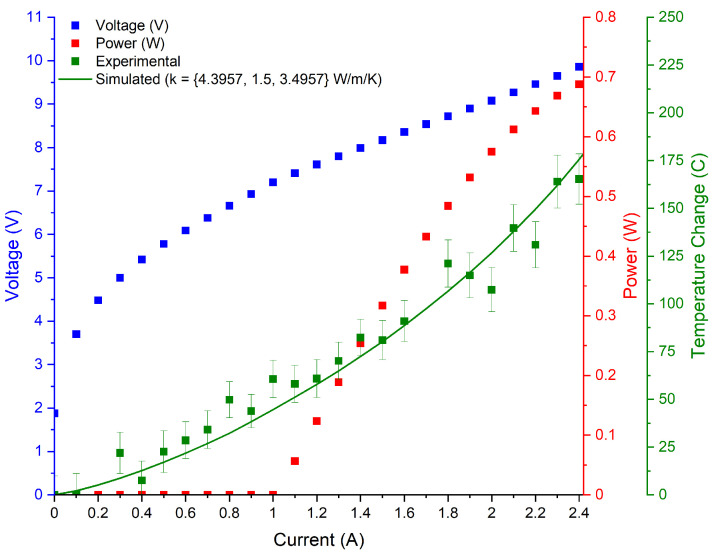
Temperature curves for the S12 device emitting at 8.7 μm. Cross-plane thermal conductivity is reported as 1.5 W/(m·K). Device dimensions are 5 mm × 9 μm.

**Figure 3:**
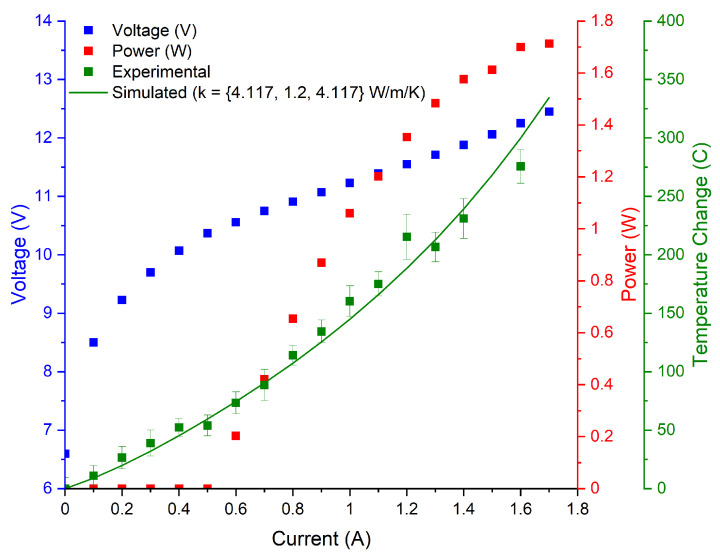
Temperature curves for the S1 device emitting at 5.7 μm. Cross-plane thermal conductivity is reported as 1.2 W/(m·K). Device dimensions are 3.15 mm × 7 μm.

**Figure 4:**
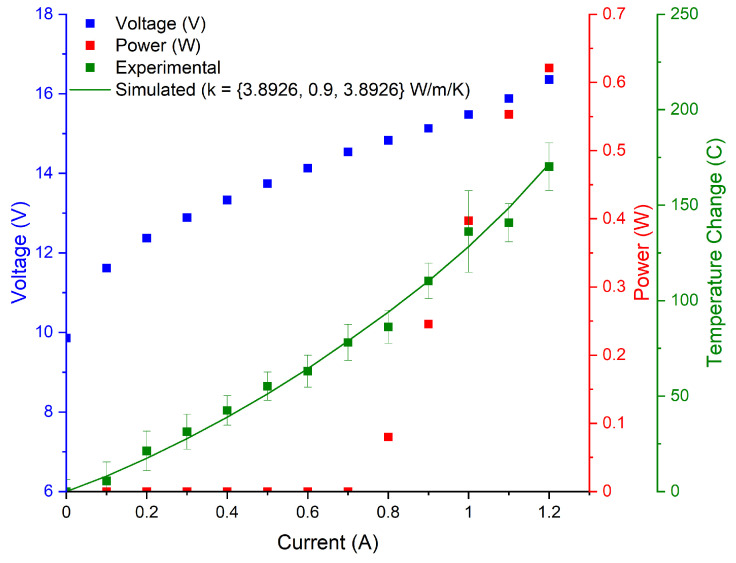
Temperature curves for S25 device emitting at 3.7 μm. Cross-plane thermal conductivity is reported as 0.9 W/(m·K). Device dimensions are 5 mm × 7.2 μm.

**Figure 5:**
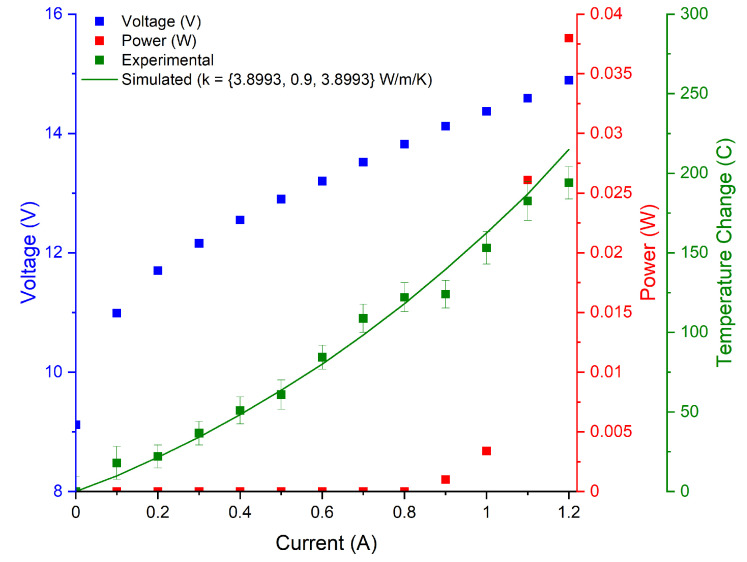
Temperature curves for D41 device also emitting at 3.7 μm. Cross-plane thermal conductivity is reported as 0.9 W/(m·K), same as the S25 device. Device dimensions are 5 mm × 8 μm.

The LIV curves, as well as the experimental and simulated temperature curves collected for the tested devices are illustrated in Figure 2, Figure 3, Figure 4 and Figure 5. The 3D COMSOL model extracted the maximum temperature at the facet for comparison with the experimentally determined facet temperature, and the COMSOL data whose thermal conductivity best agreed with the experimental temperature curves were reported. The error bars represent the uncertainty of the fit.

The S12 device was coated and tested immediately upon cleaving to avoid additional heating from optical reabsorption caused by facet oxidization [15]. As a consequence, there was no change in heating rate with change in current at the laser threshold observed in ref. [15]. The S12 device was the only LWIR device tested, and it had the highest thermal conductivity out of all the structures. This experimentally determined value fell between the estimations from the DMM and AMM.

The S1 structure has a QCL design with record efficiency [10], and it was found to match the DMM model the best out of all structures tested.

**Figure 6:**
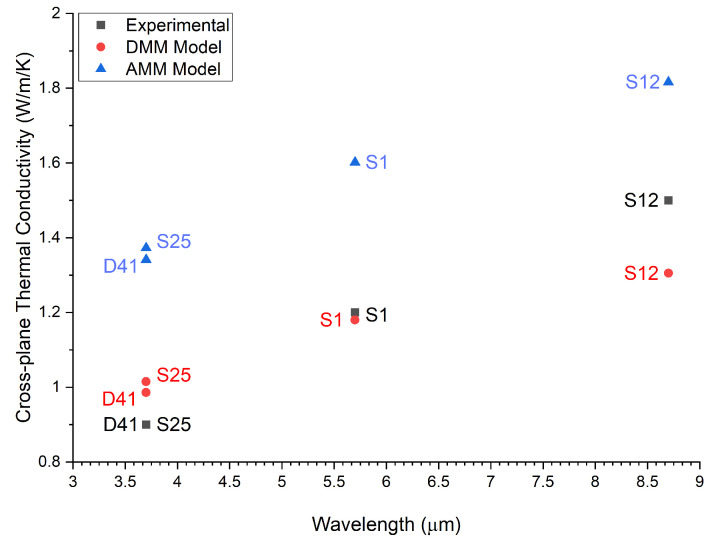
Cross-plane thermal conductivities vs wavelength for the four structures analyzed, both experimentally and through both phonon transport model calculations.

The final two devices tested, despite emitting at the same wavelength, had slightly varied active region design. These two devices were measured to have roughly the same thermal conductivity, although the model illustrated the slightly higher conductivity from S25, likely due to its reduced interface density. Both devices were measured to be slightly below the estimation given by the DMM. Figure 6 summarizes the results of the experimental, DMM, and AMM thermal conductivities.

All three analyses demonstrated the anticipated trend, and the experimental data most closely matched the DMM, which pointed to the superlattice interfaces experiencing primarily diffuse scattering. Overall, the experimental data were in good agreement with the DMM. The S12 temperature curves reported a ~10% higher conductivity when compared with the DMM, 1.5 W/(m·K). This suggested that the interface of the S12 structure was best represented by some combination of the two models. This was consistent with earlier analyses as in [2,20]. The correlation between conductivity and wavelength was clearly represented by the experimental results, which themselves also agreed with the theoretical calculations.

## 4. Discussion

**Figure 7:**
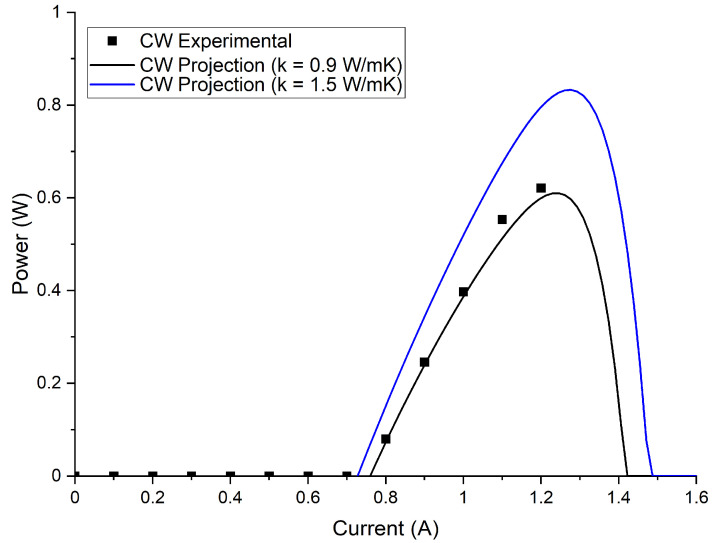
CW Power projection for the two extremes of thermal conductivity for the S25 structure.

The dependence of the conductivity on the wavelength cannot be ignored in laser design. To illustrate this, we modeled the CW LIV properties of the S25 device using the two extreme values for thermal conductivity (i.e., 1.5 W/(m·K) and 0.9 W/(m·K)). The numerical model is described in [21]. From Figure 7 we can observe that modeling the device optical power using the measured thermal conductivity, 0.9 W/m/K, gave good agreement with the experimental LI curve, while the power projection taken from a thermal conductivity of 1.5 W/(m·K) overestimated maximum power by 30%. The discrepancy would be even larger for wider devices as relative importance of cross-plane thermal conductivity for heat dissipation is higher these cases.

## 5. Conclusions

As reflected both by the model and experimental results, QCL design parameters for reduced emission wavelengths coincide with a reduced thermal conductivity in the cross-plane direction. The cross-plane thermal conductivity can be determined by a combination of Raman thermometry and thermal simulations. Over a range of QCL structures, there is a clear correlation between wavelength and thermal conductivity. The thermal model we utilize also agrees with the experimental analysis and allows for a good estimate of a structure’s thermal conductivity based on a few basic parameters. We demonstrate that the density of interfaces is the limiting factor for thermal conductivity, and by extension CW performance, in short-to-long wavelength QCLs. We show that the DMM can be used to estimate the cross-plane thermal conductivity for a given QCL structure based on AlInAs/InGaAs material composition. The observed changes in thermal conductivity for short-wavelength QCLs have a considerable impact on projected QCL performance and should be considered in QCL design.

## Figures and Tables

**Table 1:** Material parameters of samples in this study.

Structure	Wavelength (µm)	Number of Interfaces	Total Stage Thickness (Å)	Laser Core Dimensions	Barrier Composition/Total Thickness (Å)	Quantum Well Composition/Total Thickness (Å)
D41	3.7	20	427	5 mm × 8 µm	$Al_{0.78}In_{0.22}As$ 188	$Ga_{0.24}In_{0.76}As$ 239
S25	3.7	20	447	5 mm × 7.2 µm	$Al_{0.78}In_{0.22}As$ 198	$Ga_{0.23}In_{0.77}As$ 249
S1	5.7	18	453	3.15 mm × 7 µm	$Al_{0.78}In_{0.22}As$ 160	$Ga_{0.37}In_{0.63}As$ 293
S12	8.7	16	449	5 mm × 9 µm	$Al_{0.65}In_{0.35}As$ 115	$Ga_{0.41}In_{0.59}As$ 334

**Table 2:** Composition-dependent material parameters for TBR analysis.

Parameter	$\mathrm{Material}\;1\;(Al_{x}In_{1-x}As){\;}^{1}$	$\mathrm{Material}\;2\;(Ga_{x}In_{1-x}As){\;}^{2}$
$\mathrm{Elastic}\;\mathrm{Constant}\;C_{11}\left[\frac{N}{m^{2}}\right]\;$	(8.34 + 3.68x) × 10^10^	(8.34 + 3.56x) × 10^10^
$\mathrm{Elastic}\;\mathrm{Constant}\;C_{44}\left[\frac{N}{m^{2}}\right]$	(3.95 + 1.94x) × 10^10^	(3.95 + 2.01x) × 10^10^
$\mathrm{Material}\;\mathrm{Density}\;\rho \left[\frac{kg}{m^{3}}\right]\;$	5680 − 1920x	5680 − 370x
$\mathrm{Longitudinal}\;\mathrm{Acoustic}\;\mathrm{Wave}\;\mathrm{Speed}\;\nu_{L}\left[\frac{m}{s}\right]$	$\sqrt{\frac{C_{11}}{\rho }}\;$	
$\mathrm{Transverse}\;\mathrm{Acoustic}\;\mathrm{Wave}\;\mathrm{Speed}\;\nu_{T}\left[\frac{m}{s}\right]$	$\sqrt{\frac{C_{44}}{\rho }}$	
$\mathrm{Debye}\;\mathrm{Temperature}\;\Theta_{D}\left[K\right]$	280 + 166x	280 + 110x
$\mathrm{Bulk}\;\mathrm{Thermal}\;\mathrm{Conductivity}\;k\left[\frac{W}{mK}\right]$	2.5	5.0

^1^ Interpolated from material parameters of InAs, InAlAs and AlAs taken from [6,8,11,20]. ^2^ Taken from [6,8,11,20].

**Table 3:** Results of calculation of cross-plane conductivity and TBR using the DMM and AMM.

Wavelength (µm)	Interface Density N/L (nm^−1^)	DMM Thermal Boundary Resistance (m^2^K/W)	AMM Thermal Boundary Resistance (m^2^K/W)	DMM Cross-Plane Thermal Conductivity (W/m/K)	AMM Cross-Plane Thermal Conductivity (W/m/K)
3.7	0.468	1.549 × 10^−9^	9.765 × 10^−9^	0.986	1.341
3.7	0.447	1.557 × 10^−9^	9.83 × 10^−9^	1.015	1.373
5.7	0.397	1.452 × 10^−9^	8.9 × 10^−9^	1.18	1.602
8.7	0.356	1.445 × 10^−9^	8.4 × 10^−9^	1.305	1.816

**Table 4:** Values for each term in cross-plane thermal conductivity calculation.

Wavelength (µm)	$\frac{L_{1}}{L}k_{1}^{-1}\left[\frac{mK}{W}\right]$	$\frac{L_{2}}{L}k_{2}^{-1}\left[\frac{mK}{W}\right]$	$\frac{N}{L}R^{\left(ave\right)}\left[\frac{mK}{W}\right]$
3.7	0.176	0.112	0.726
3.7	0.177	0.111	0.697
5.7	0.141	0.13	0.577
8.7	0.102	0.149	0.515

## Data Availability

Data supporting reported results can be found in this article.
